# Clinical utility of expanded carrier screening: results-guided actionability and outcomes

**DOI:** 10.1038/s41436-018-0321-0

**Published:** 2018-10-11

**Authors:** Katherine A. Johansen Taber, Kyle A. Beauchamp, Gabriel A. Lazarin, Dale Muzzey, Aishwarya Arjunan, James D. Goldberg

**Affiliations:** Counsyl, Inc., South San Francisco, CA USA

**Keywords:** expanded carrier screening, prenatal diagnosis, pregnancy management, clinical utility, at-risk couple.

## Abstract

**Purpose:**

Expanded carrier screening (ECS) informs couples of their risk of having offspring affected by certain genetic conditions. Limited data exists assessing the actions and reproductive outcomes of at-risk couples (ARCs). We describe the impact of ECS on planned and actual pregnancy management in the largest sample of ARCs studied to date.

**Methods:**

Couples who elected ECS and were found to be at high risk of having a pregnancy affected by at least one of 176 genetic conditions were invited to complete a survey about their actions and pregnancy management.

**Results:**

Three hundred ninety-one ARCs completed the survey. Among those screened before becoming pregnant, 77% planned or pursued actions to avoid having affected offspring. Among those screened during pregnancy, 37% elected prenatal diagnostic testing (PNDx) for that pregnancy. In subsequent pregnancies that occurred in both the preconception and prenatal screening groups, PNDx was pursued in 29%. The decision to decline PNDx was most frequently based on the fear of procedure-related miscarriage, as well as the belief that termination would not be pursued in the event of a positive diagnosis.

**Conclusion:**

ECS results impacted couples’ reproductive decision-making and led to altered pregnancy management that effectively eliminates the risk of having affected offspring.

## Introduction

Serious recessive and X-linked conditions affect an estimated 1 in 300 pregnancies.^1^ However, universal screening for only the two conditions recommended by current guidelines, cystic fibrosis (CF) and spinal muscular atrophy,^2–4^ misses nearly 70% of carriers of rare disease^5^ and fails to detect between 13% and 94% of pregnancies affected with profound and severe conditions, depending on ethnicity.^6,7^ Expanded carrier screening (ECS), i.e., testing reproductive partners’ carrier status for a large number of recessive and X-linked conditions without regard to ethnicity, addresses this gap. When ECS is undertaken during the preconception period, results enable interventions to avoid affected pregnancies, such as in vitro fertilization (IVF) with preimplantation genetic testing for monogenic conditions (PGT-M); and when undertaken during the prenatal period, results facilitate prenatal diagnostic testing (PNDx; amniocentesis or chorionic villus sampling, CVS) and pregnancy management (including termination). The American College of Obstetricians and Gynecologists (ACOG) recognizes ECS as an acceptable strategy for carrier screening,^8^ and together with American College of Medical Genetics and Genomics, the Society for Maternal–Fetal Medicine, the National Society of Genetic Counselors, and the Perinatal Quality Foundation, acknowledges the unique benefits and considerations of ECS.^9^

For certain conditions, population-wide carrier screening has well-established clinical utility, i.e., the improvement in health outcomes as a result of preconception screening, prenatal diagnosis, and early identification of affected pregnancies that enables condition-specific counseling and management.^9^ Between 1970 and 2000, screening for Tay–Sachs disease carrier status reduced the incidence of Tay–Sachs disease in the US and Canadian Ashkenazi Jewish population by 90%.^10,11^ Similarly, the prevalences of CF- and thalassemia-affected births were reduced in other countries and in parts of the United States following the institution of carrier screening programs.^12–15^

Although ECS has been in existence for nearly a decade, evidence of its clinical utility has only recently emerged. In a study of 64 at-risk couples (ARCs; defined as a reproductive couple in which both individuals carry pathogenic variants in the same gene, or a female carries an X-linked pathogenic variant) identified through ECS, 76% of those at risk for severe or profound conditions took or planned to take action to reduce the risk of an affected birth, including IVF with PGT-M and PNDx.^16^ In a separate study conducted among couples undergoing IVF, all ARCs in the sample (8/8) underwent or planned to undergo PGT-M to avert an affected birth.^17^ Though conducted on cohorts with relatively few ARCs, these studies suggest that ECS enables reproductive decision-making that reduces the risk of having affected offspring, potentially leading to reduced incidence for a broad range of screened conditions and in diverse populations.

To characterize in more detail the clinical utility of ECS, we studied the actions taken by nearly 400 ARCs after receiving ECS results for up to 176 conditions. Results demonstrate that more than three-quarters of ARCs screened preconceptionally planned or pursued actions that reduced the risk of having affected offspring, and more than one-third of ARCs screened prenatally underwent PNDx to inform pregnancy management, providing further evidence that ECS guides reproductive decision-making and impacts pregnancy outcomes.

## Materials and methods

### Cohort generation

To generate a survey cohort, data for more than 270,000 individuals who had received ECS from Counsyl (Family Prep Screen or Foresight^TM^ Carrier Screen) between 1 September 2015 and 31 December 2017 were queried for females who (1) were found to be carriers of a pathogenic or likely pathogenic variant conferring risk for at least one of 176 autosomal recessive or X-linked conditions currently included in Counsyl’s Foresight ECS,^1^ (2) were aged 18 years or older, (3) had consented to being contacted about participating in research at Counsyl, and (4) for those carrying pathogenic or likely pathogenic variants associated with autosomal recessive conditions, had reproductive partners meeting the same eligibility criteria and who were confirmed by Counsyl as being carriers of a pathogenic variant in the same gene. Couples carrying only variants known to cause mild presentations of biotinidase deficiency (D444H), NPHS2-related nephrotic syndrome (R229Q), and 21-OH deficient congenital adrenal hyperplasia (CAH) (*CYP21A2* gene duplication) were excluded.

The resulting cohort was validated via software to ensure that an email address was on file for the female member of each ARC, and that the email address did not appear twice in the cohort as that could indicate a female having more than one male reproductive partner and thus constituting more than one ARC. Further, inclusion criteria for 40 randomly selected individuals in the cohort were verified by Counsyl staff not involved in the study as a quality-control check of the software-directed validation. After final validation, the cohort invited to participate comprised 1701 ARCs whose current or future pregnancies were at risk for 78 conditions in aggregate.

### Survey development

**Fig. 1:**
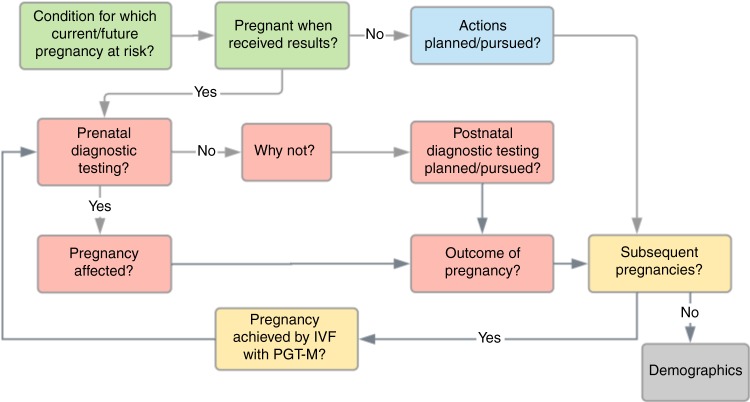
**Survey flow.** Survey questions were divided into five sections, denoted by color: foundational information such as condition(s) for which respondents were found to be carriers and pregnancy status at the time of receiving results (green); actions planned or pursued by those receiving expanded carrier screening (ECS) results before pregnancy (blue); actions pursued by those receiving ECS results during pregnancy (pink); actions pursued in subsequent pregnancies (yellow); and demographic information (gray).

Survey questions were developed by reviewing and expanding on a previously published survey of ARCs.^16^ Questions were also reviewed by two pediatric geneticists not affiliated with Counsyl to ensure the accuracy of clinical content. Questions were divided into five sections, as indicated in Fig. 1, and are available in Supplementary Figure 1.

Survey questions were programmed into commercial software (Logician®, Decision Analyst Inc., Arlington, TX) to eliminate logic errors, prevent omissions, define acceptable and unacceptable answer codes, and build in skip patterns; and to provide the web-based platform for response collection. Questions were pretested with four ARCs to determine understandability, appropriate wording, completion of questions as intended, and approximate time to complete the survey. Individual ARCs were observed as they navigated through the online survey, and revisions were made based on their feedback.

### Survey fielding

The survey was fielded by Decision Analyst, Inc. between 28 February 2018 and 19 March 2018. Female members of 1701 ARCs were invited by email to participate. After invitations were sent, 42 emails were undeliverable, likely due to incorrect email addresses on file or email accounts that were no longer in use. This effectively reduced the cohort to 1659 ARCs. To complete the survey, respondents were directed to a Decision Analyst, Inc.–hosted online survey site, which included an explanation of the research project and asked for consent to participate. A total of four reminders were sent to nonresponders over the course of the 19-day survey period. Those who completed the survey after the initial invitation or first reminder were eligible to receive a $30 incentive or to donate $30 to charity. This incentive was increased to $55 with the second, third, and fourth reminders.

### Data analysis

Data management and tabulation were accomplished via UNCLE® (Hermosa Beach, CA) and analysis performed by SPSS (IBM, Armonk, NY). Descriptive statistics were used to characterize general data trends. Statistical significance between proportions was determined using chi-square analysis; a result was considered significant when *p* < 0.05 at the 95% confidence level. Confidence intervals (CIs) were determined using the Jeffreys method.^18^ To compare actions among ARCs by disease severity, conditions were categorized by severity according to the method described by Lazarin et al.^7^ For ARCs reporting that their pregnancies were at risk for more than one condition, the category corresponding to the more severe condition was used. Couples indicating that their pregnancies were at risk for CAH were placed into the moderate risk category because no distinction was made between classic and nonclassic CAH.

### Institutional review board approval

This study was reviewed and designated as exempt by Western Institutional Review Board.

## Results

### Cohort characteristics

Three hundred ninety-one respondents completed the survey for an overall response rate of 24%. Ninety-five percent of respondents were between the ages of 25 and 44 years, and 39% were pregnant when they received their ECS results, with the remainder not pregnant (60%) or preferring not to indicate pregnancy status (0.5%) (Table 1). Among those who were not pregnant, 54% were undergoing or planning to undergo IVF at the time they received their ECS results. Respondents were geographically dispersed, and along with their reproductive partners, represented more than 15 ethnicities and more than nine religions (Table 1). ARCs reported being at risk for pregnancies affected by 53 different conditions, with profound, severe, and moderate conditions represented (Supplementary Table 1). Ten percent and 1.8% of respondents reported being at risk for pregnancies affected by two or three conditions, respectively (Supplementary Table 1).

**Table 1:** Respondent demographics

Characteristics	Total Respondents, n (%)	
Total Respondents	391 (100)	
Age of female partner^a^		
18–24	14 (3.6)	
25–34	211 (54)	
35–44	160 (41)	
45+	6 (1.5)	
Pregnant when received results^a^		
Yes	154 (39)	
0–13 weeks pregnant	72 (47)	
14–26 weeks pregnant	74 (48)	
27 or more weeks pregnant	5 (3.2)	
Did not answer/Did not recall	3 (1.9)	
No	235 (60)	
Planning or undergoing IVF when received results^b^	122 (54)	
Prefer not to say	2 (0.5)	
Geographic region^a^		
Northeast	85 (22)	
Midwest	50 (13)	
South	125 (32)	
West	128 (33)	
Outside U.S.	3 (0.8)	
Ethnicity^c^	Female Partner	Male Partner
Northern European	119 (30)	113 (29)
Other/Mixed Caucasian	114 (29)	107 (27)
Ashkenazi Jewish	75 (19)	70 (18)
Southern European	34 (8.7)	38 (9.7)
East Asian	34 (8.7)	24 (6.1)
Hispanic	19 (4.9)	28 (7.4)
South Asian	16 (4.1)	17 (4.3)
African or African American	14 (3.6)	17 (4.3)
Southeast Asian	11 (2.8)	9 (2.3)
Middle Eastern	9 (2.3)	10 (2.6)
French Canadian or Cajun	9 (2.3)	7 (1.8)
Native American	3 (0.8)	5 (1.3)
Pacific Islander	0 (0)	2 (0.5)
Other	3 (0.8)	1 (0.3)
Unknown	2 (0.3)	3 (0.8)
Prefer not to say	11 (2.8)	13 (3.3)
Religion^a^	Female Partner	Male Partner
No religious affiliation	89 (23)	101 (26)
Jewish	70 (18)	62 (16)
Protestant	67 (17)	55 (14)
Catholic	65 (17)	67 (17)
Agnostic	26 (6.6)	66 (17)
Atheist	11 (2.8)	23 (5.9)
Hindu	7 (1.8)	6 (1.5)
Buddhist	6 (1.5)	3 (0.8)
Muslim	5 (1.3)	7 (1.8)
Mormon	3 (0.8)	5 (1.3)
Other	17 (4.3)	15 (3.8)
Prefer not to say	25 (6.4)	25 (6.4)

*IVF*: In vitro fertilization.

^a^Percents sum to just under or over 100% due to rounding.

^b^8 ARCs did not answer whether they were undergoing IVF at the time of ECS; percent is therefore calculated out of 227.

^c^Percents sum to greater than 100% because respondents could pick more than one answer.

### Actions taken or planned as a result of preconception screening

Of respondents screened preconceptionally (Fig. 1, blue), 77% reported planning or pursuing actions that impact pregnancy management and/or reduce the risk of an affected pregnancy (Table 2). These included IVF with PGT-M (59%), PNDx (by amniocentesis or CVS) (20%), use of a donor gamete (7.7%), adoption (5.1%), and no longer planning to get pregnant (3.8%). Preconception respondents planned or pursued other actions that do not directly affect pregnancy management: once pregnant, inform other doctors of the risk for the condition (29%, *n* = 69); and test children or other family members for the condition (15%, *n* = 36) (not shown). Only 4.6% of respondents (*n* = 11) did not plan or pursue any action (not shown).

**Table 2:** Actions planned or pursued by ARCs screened during the preconception period

	All Severities, n (%; CI)	Profound, n (%; CI)	Severe, n (%; CI)	Moderate, n (%; CI)	Severity Unassigned,^a^ n (%; CI)
Screened Preconceptionally	235 (100)	34 (14)	153 (65)	34 (14)	14 (5.9)
Planned/pursued any of the following actions:^b^	180 (77; 71–82)	31 (91; 78–97)^c^	118 (77; 70–83)	22 (65; 47–79)^c^	9 (64; 38–85)
IVF with PGT-M	139 (59; 53–65)	23 (68; 51–81)	92 (60; 52–68)	20 (59; 42–74)	4 (29; 11–55)
PNDx	48 (20; 16–26)	8 (24; 12–40)	31 (20; 14–27)	6 (18; 8–33)	3 (21; 6–47)
Donor gamete	18 (7.7; 5–12)	4 (12; 4–26)	11 (7.2; 4–12)	1 (2.9; 0–13)	2 (14; 3–38)
Adoption	12 (5.1; 3–8)	3 (8.8; 3–22)	9 (5.9; 3–10)	0 (0; 0–7)	0 (0; 0–16)
No longer planning to get pregnant	9 (3.8; 2–7)	1 (2.9; 0–13)	6 (3.9; 2–8)	1 (2.9; 0–13)	1 (7; 0.7–29)

*ARC*: At-risk couple. *CI*: confidence interval, 95%. *IVF*: In vitro fertilization. *PGT-M*: Preimplantation genetic testing for monogenic conditions. *PNDx*: Prenatal diagnostic testing.

^a^These ARCs did not recall, were not clear about, or did not answer the condition for which their future pregnancies were at risk and therefore could not be not assigned to severity classifications.

^b^Respondents could choose more than one option, so percents of individual actions could sum to greater than 100.

^c^Difference between Profound and Moderate is statistically significant (p=0.008).

When stratified by condition severity,^7^ the proportion of respondents planning or pursuing actions was highest among those whose future pregnancies were at risk for a profound condition (91%), followed by a severe condition (77%), and a moderate condition (65%) (Table 2). However, only the difference between the profound and moderate groups was statistically significant at the 95% confidence level (*p* = 0.008). This pattern was present for each of the actions except for no longer planning a pregnancy (Table 2), but no other differences were found to be statistically significant.

### Diagnostic testing after prenatal screening

Of respondents screened prenatally (Fig. 1, pink), 37% reported having undergone PNDx (by amniocentesis or CVS) (Table 3). When stratified by condition severity, the proportion of respondents having undergone PNDx was highest for pregnancies at risk for profound conditions (47%), followed by severe (38%) and moderate (29%), but differences were not statistically significant (Table 3). Of pregnancies that underwent PNDx, 36% were found to be affected; 40% of affected pregnancies were terminated (Table 3). Conditions for which pregnancies were found to be affected are listed in Supplementary Table 2.

**Table 3:** Actions and outcomes of ARCs screened during the prenatal period

	All Severities, n (%; CI)	Profound, n (%; CI)	Severe, n (%; CI)	Moderate, n (%; CI)	Severity Unassigned,^a^ n (%; CI)
Screened Prenatally	154 (100)	15 (9.7)	104 (68)	28 (18)	7 (4.5)
Underwent PNDx	56 (37; 29–35)^b^	7 (47; 24–71)	40 (38; 30–49)	8 (29; 15–47)	1 (14; 1.6–50)
Pregnancies affected	20 (36; 23–48)^c^	4 (57; 23–86)	11 (28; 16–43)^d^	4 (50; 20–80)	1 (100; 15–100)
Pregnancy outcome:^e^					
Terminated	8 (40; 21–62)	2 (50; 12–88)	5 (45; 20–73)	1 (25; 3–72)	0 (0; 0–85)
Live birth	8 (40; 21–62)	1 (25; 3–72)	5 (45; 20–73)	2 (50; 12–88)	0 (0; 0–85)
Not born yet	3 (15; 4–35)	1 (25; 3–72)	0 (0; 0–26)	1 (25; 3–72)	1 (100; 15–100)
Stillborn	1 (5.0; 0.5–21)	0 (0; 0–49)	1 (9.1; 1–35)	0 (0; 0–49)	0 (0; 0–85)
Did not undergo PNDx	95 (63; 55–70)^b^	8 (53; 29–76)	62 (60; 50–69)	19 (68; 49–82)	6 (86; 50–98)
Pregnancy outcome:					
Live birth	71 (75; 70–86)	7 (88; 55–99)	47 (76; 64–85)	14 (74; 52–89)	3 (50; 17–83)
Planned/pursued postnatal diagnosis	44 (62; 50–73)	1 (14; 2–50)^f^	29 (62; 47–75)^f^	13 (93; 71–99)^f^	1 (33; 3.9–82)
Not born yet	20 (21; 14–30)	0 (0; 0–26)	14 (23; 14–35)	4 (21; 7.6–43)	2 (33; 7.7–71)
Miscarried	2 (2.1; 0–7)	0 (0; 0–26)	1 (1.6; 0–7)	1 (5.3; 0.6–22)	0 (0; 0–33)
Terminated	2 (2.1; 0–7)	1 (13; 1–45)	0 (0; 0–4)	0 (0; 0–12)	1 (17; 1.9–55)

*ARC*: At-risk couple. *CI*: Confidence interval, 95%. *PNDx*: Prenatal diagnostic testing.

^a^These ARCs did not recall, were not clear about, or did not answer the conditions for which their pregnancies were at risk and therefore could not be categorized by severity.

^b^Out of 154 ARCs screened prenatally, 3 did not indicate if they underwent PNDx. Percent is therefore calculated out of 151 screened prenatally.

^c^1 ARC was still awaiting results. Percent is therefore calculated out of 55 pregnancies that underwent PNDx.

^d^1 ARC was still awaiting results. Percent is therefore calculated out of 39 pregnancies at risk for severe conditions that underwent PNDx.

^e^Conditions with which pregnancies were found to be affected are indicated in Supplementary Table 3.

^f^Differences are statistically significant at the 95% confidence level: profound vs. severe: p=0.02, severe vs. moderate: p=0.03, profound vs. moderate: p=0.0003.

Respondents screened prenatally who did not undergo PNDx and whose pregnancies resulted in a live birth were asked if they had pursued diagnostic testing after the baby’s birth or planned to do so in the near future; 62% answered in the affirmative (Table 3). When stratified by severity, the inverse pattern of that seen for PNDx was observed, i.e., the proportion of respondents who pursued or planned postnatal diagnosis was smallest for pregnancies at risk for profound conditions (14%), followed by severe (62%) and moderate (93%) (Table 3). These differences were statistically significant at the 95% confidence level (profound vs. severe: *p* = 0.02, severe vs. moderate: *p* = 0.03, profound vs. moderate: *p* = 0.0003).

Respondents screened prenatally who did not undergo PNDx were asked the reason(s) that they chose not to undergo such testing. Top reasons cited were to avoid the increased risk of miscarriage associated with amniocentesis and CVS (35%), that results would not have led to pregnancy termination (27%), and a perception that the risk of an affected pregnancy was low (26%) (Supplementary Table 3).

### Actions taken in subsequent pregnancies

ARCs screened both before and during pregnancy were asked to report actions undertaken for pregnancies conceived subsequent to receiving ECS results. These are pregnancies conceived after those screened preconceptionally received their results, and pregnancies conceived after the one during which those screened prenatally received their results (Fig. 1, yellow). One hundred twenty-six and 40 subsequent pregnancies were reported by ARCs screened preconceptionally and prenatally, respectively (Table 4). Among all subsequent pregnancies, 35% were achieved by IVF with PGT-M. ARCs screened before becoming pregnant were significantly more likely to achieve subsequent pregnancies by undergoing IVF with PGT-M (40%) than were those screened during a previous pregnancy (20%) (*p* = 0.02) (Table 4).

**Table 4:** Actions and outcomes of pregnancies occurring subsequent to ECS test results

	Total, n (%, CI)	Screened before becoming pregnant, n (%, CI)	Screened during previous pregnancy, n (%, CI)
Subsequent pregnancies	166 (100)	126 (76)	40 (24)
Achieved by IVF with PGT-M	58 (35; 28–42)	50 (40; 31–48)^a^	8 (20; 10–34)^a^
Underwent PNDx	48 (29; 22–36)	37 (29; 22–38)	11 (28; 16–34)
Pregnancies affected	12 (29; 17–44)^b^	11 (34; 20–52)^c^	1 (11; 1–41)^d^
Pregnancy outcome:^e^			
Terminated	9 (75; 47–92)	8 (73; 43–92)	1 (100; 15–100)
Live birth	3 (25; 8–53)	3 (27; 8–57)	0 (0; 0–85)
Did not undergo PNDx	118 (71; 51–69)	89 (71; 62–78)	29 (72; 57–84)
Pregnancy outcome:^f^			
Live birth	51 (44; 35–53)	42 (47; 37–58)	9 (32; 17–51)
Planned/pursued postnatal diagnosis	23 (45; 32–59)	20 (48; 33–62)	4 (44; 17–45)
Not born yet	43 (37; 28–46)	28 (31; 23–42)	15 (54; 36–71)
Miscarried	20 (17; 11–25)	17 (19; 12–28)	3 (11; 3–26)
Terminated	3 (2.6; 1–7)	2 (2.2; 0–7)	1 (3.6; 0–16)

*CI*: Confidence interval, 95%. *IVF*: In vitro fertilization. *PGT-M*: Preimplantation genetic testing for monogenic conditions. *PNDx*: Prenatal diagnostic testing.

^a^Difference is statistically significant at 95% confidence level (p=0.02).

^b^7 of 48 that underwent PNDx are still waiting on the results. Percent affected is therefore calculated out of 41.

^c^5 of 37 that underwent PNDx are still waiting on results. Percent affected is therefore calculated out of 32.

^d^2 of 11 that underwent PNDx are still waiting on results. Percent affected is calculated out of 9.

^e^Conditions for which pregnancies were found to be affected are indicated in Supplementary Table 3.

^f^1 of 29 screened during a previous pregnancy that did not undergo PNDx did not indicate pregnancy outcome. Percents are therefore calculated out of 117 (total pregnancies that did not undergo PNDx) or 28 (screened during a previous pregnancy and did not undergo PNDx).

Nearly one-third (29%) of subsequent pregnancies underwent PNDx; no significant difference in this proportion was found among those screened before becoming pregnant (29%) versus those screened during a previous pregnancy (28%) (Table 4). The proportion that underwent PNDx in a subsequent pregnancy also was not significantly different than the proportion that underwent PNDx in the pregnancy during which ECS results were received (37%; Table 3). Among subsequent pregnancies that underwent PNDx, 29% were found to be affected; 75% of affected pregnancies were terminated (Table 4). Conditions for which pregnancies were found to be affected are listed in Supplementary Table 2. Taken together with pregnancies during which results were received, the termination rate for affected pregnancies was 53% (Supplementary Table 2).

ARCs who reported subsequent pregnancies and did not undergo PNDx were asked the reason(s) that they chose not to undergo such testing. Top reasons cited were that it was not necessary because the pregnancy was achieved by IVF with PGT-M (28%), a perception that the risk of an affected pregnancy was low (17%), to avoid the increased risk of miscarriage associated with amniocentesis and CVS (14%), the pregnancy miscarried before testing could be performed (14%), and that results would not have led to pregnancy termination (10%) (Supplementary Table 3). Compared with reasons cited by those who were pregnant when they received their screening results, significantly fewer cited the risk of miscarriage (*p* = 0.0005) and an unwillingness to terminate the pregnancy (*p* = 0.001) as reasons for not undergoing PNDx during a subsequent pregnancy (Supplementary Table 3). Of respondents who did not undergo PNDx and whose subsequent pregnancies resulted in a live birth, 45% planned or pursued diagnostic testing after the baby’s birth (Table 4).

## Discussion

Our study describes the clinical utility of ECS among the largest cohort of ARCs studied to date. It examined actions of a geographically, ethnically, and religiously diverse cohort of couples screened for up to 176 conditions both in the preconception and prenatal stages. Because Mendelian diseases are rare, past studies of ARC behavior have examined small cohorts. For example, in a study of more than 3700 couples receiving ECS in a fertility clinic, only 8 ARCs were identified;^17^ and in a sample of over 100,000 couples who received ECS, 537 ARCs were identified, only 64 of whom participated in the outcomes portion of the study.^16^ Our study of 391 ARCs—gathered from more than 270,000 total individuals who had undergone ECS—therefore makes a substantial contribution to the evidence base supporting ECS as an impactful tool for reproductive decision-making.

Widespread clinical adoption and insurance coverage of health-care interventions often rely on demonstrations of clinical utility.^19^ In the context of genetic testing, clinical utility is defined based on the value of the test result: information that leads to an improved health outcome, including diagnosis, treatment, management, or disease prevention, that will benefit a patient or his/her family members.^20^ Our study demonstrates the clinical utility of ECS: more than three-quarters of ARCs in the study cohort who were tested preconceptionally planned or took action to avert an affected pregnancy (IVF with PGT-M, use of donor gametes, adoption, or avoidance of pregnancy) (Table 2), and more than one-third in the study cohort who were tested prenatally took action to establish a prenatal diagnosis (Table 3). ARCs in our study terminated more than half of affected pregnancies, demonstrating the substantial impact of ECS results on pregnancy management. Among the study cohort, more than one-third of the pregnancies conceived subsequent to receiving ECS results were achieved using IVF with PGT-M (Table 4), effectively preventing an affected pregnancy and demonstrating the utility of knowing carrier status before becoming pregnant.

The proportion of ARCs identified while pregnant and electing PNDx in this study (37%, Table 3) is consistent with that found in a previous ECS outcomes study (42%)^16^. It is also consistent with observations for other widely adopted screening tests: 39% of those with a trisomy 21 positive noninvasive prenatal screen and 45% of those with a trisomy 21 positive maternal serum screen elect to undergo invasive diagnostic testing.^21,22^ Among pregnant women who decline amniocentesis or CVS following a trisomy 21 positive maternal serum screen, the risk of procedure-related miscarriage is the most commonly cited reason.^21^ We found the same: among ARCs receiving ECS results when they were pregnant, the most frequently cited reason for not pursuing PNDx was the risk of miscarriage (Supplementary Table 3). ARCs in our study also reported that they did not undergo PNDx because they would not have pursued pregnancy termination in the event of a positive result. As actionability of a positive result extends beyond pregnancy termination to include altered or enhanced pregnancy management, rapid diagnosis of a neonate suspected to be affected, and immediate intervention or treatment after birth, this result suggests that patients could benefit from education and/or genetic counseling that explains the range of benefits of establishing a diagnosis prenatally.

In pregnancies conceived subsequent to the receipt of ECS results (Fig. 1, yellow), the top reasons cited for not pursuing PNDx were different than those cited for pregnancies during which ECS results were received. ARCs most frequently believed PNDx was not necessary because the pregnancy had been achieved by IVF with PGT-M, reflecting the large number of ARCs in this group who had undergone IVF with PGT-M (Supplementary Table 3). They also believed that PNDx was not necessary because they perceived their pregnancy to be at low risk of being affected (Supplementary Table 3). Those undergoing assisted reproductive technologies are less likely to elect amniocentesis or CVS than those whose pregnancies are spontaneous^23,24^ despite guidelines recommending that couples undergoing PGT-M be counseled that confirmatory PNDx is necessary due to the technical challenges of PGT.^25^

In addition to enabling PNDx, ECS results enable targeted postnatal diagnostic testing for conditions that may otherwise be difficult to recognize and that could lead to a years-long diagnostic odyssey that includes suboptimal or ineffective treatment.^26^ In our study, a large proportion of ARCs who declined PNDx planned or had already pursued postnatal diagnostic testing (62% of those pregnant when they received results and 45% who received results prior to subsequent pregnancies) (Tables 3 and 4), suggesting that a diagnosis was established that enabled treatment or other care, or that the condition for which the pregnancy had been at risk was ruled out. Knowing that postnatal diagnostic testing is an option may diminish the imperative to undergo PNDx. Some patients may mistakenly consider newborn screening (NBS), conducted in the first few days after birth, to be sufficient to detect serious genetic disease. However, as ACOG has acknowledged, NBS does not diminish the potential benefits of carrier screening.^8^

Consistent with a previous study,^16^ our data suggest that, whether undergoing ECS preconceptionally or prenatally, the severity of the condition affected ARCs’ decisions to plan or pursue actions. This is also in line with the finding that categorizing conditions by severity is valuable to prospective parents as they consider the actions they might undertake after receiving ECS results.^27^ Importantly, ECS for select moderate severity conditions had demonstrable clinical utility: nearly two-thirds of ARCs at risk for future pregnancies with moderate severity conditions reported planning or pursuing actions (Table 2), and nearly one-third of ARCs whose pregnancy was at risk for a moderate condition elected PNDx (Table 3).

Our study had limitations that should be noted. It relied on patients to recall their actions stemming from ECS results. Patient memory is sometimes inaccurate; for example, the proportion of ARCs reporting being at risk for two or three conditions (10% and 1.8%, respectively) is higher than would have been expected based on the estimated number of such ARCs in the general population. We also cannot rule out response bias; those who took action based on ECS results may have been more willing than those who did not to report on such actions. Conversely, some invited ARCs may have declined to participate altogether given the sensitive nature of pregnancy management. Among our cohort screened preconceptionally, 54% were undergoing or planning to undergo IVF at the time that they received ECS results, suggesting that the cohort overrepresented couples who seek fertility assistance. Because IVF with PGT-M can be expensive and insurance coverage is not available to all patients, studies assessing costs of widespread implementation of ECS are needed. The cohort was also enriched with ARCs whose current or future pregnancies were at risk for conditions that are more frequent in the population, including fragile X syndrome, cystic fibrosis, and GJB2-related DFNB1 nonsyndromic hearing loss and deafness. We sought to diminish any outsized effects these conditions may have had by analyzing actions in aggregate and by condition severity.

The data here demonstrate that ECS prompts changes in pregnancy management resulting in fewer births affected with serious genetic diseases. The frequency of these changes is consistent across carrier screening studies and with data for aneuploidy screening, for which clinical utility is generally accepted. This trend suggests clinical value in screening for diseases that have historically gone undetected.

## Electronic supplementary material

Supplementary Figure 1

Supplementary Table 1

Supplementary Table 2

Supplementary Table 3
