# Supplementary material for: Clinical utility of expanded carrier screening: results-guided actionability and outcomes
Source: Genet Med. 2018 Oct 11;21(5):1041–8. doi: 10.1038/s41436-018-0321-0 (PMC6752268; doi:10.1038/s41436-018-0321-0)
Supplement: Supplementary file 1 — Supplementary Figure 1 [file 41436_2018_321_MOESM1_ESM.pdf]

Supplementary Figure 1. Survey questions as fielded.

Thank you for agreeing to complete this brief survey. Your individual answers will be anonymous and strictly confidential.

S1. What is your sex?
(Choose One Answer)

1 Male
2 Female

S2. What is your age?
(Please Type A Whole Number In The Box Below)

S3. Did you or your partner undergo carrier screening within the last 3 years? As a reminder, carrier screening is a test that determines whether you or your partner carry a gene variation that could be passed on to your children, increasing their risk for certain diseases.
(Choose One Answer)

1 Yes
2 No
3 Don't recall

If "Yes" on S3:
Do you agree to participate in this confidential survey?
(Choose One Answer)

1 Yes
2 No

Q1. For what condition(s) were you told that your pregnancy or future pregnancy was at increased risk?

1 Alpha thalassemia
2 Aspartylglycosaminuria
3 Ataxia-telangiectasia
4 ATP7A-related disorders (including Menkes syndrome and Occipital Horn syndrome)
5 Bardet-Biedl syndrome, BBS1-related
6 Bardet-Biedl syndrome, BBS10-related
7 Biotinidase deficiency
8 Canavan disease
9 Carnitine palmitoyltransferase IA deficiency
10 Carnitine palmitoyltransferase II deficiency
11 Citrullinemia type 1
12 COL4A4-related Alport syndrome
13 Congenital adrenal hyperplasia (classic or non-classic)
14 Congenital disorder of glycosylation type Ia
15 Congenital disorder of glycosylation type Ic
16 Congenital Finnish nephrosis
17 Cystic fibrosis
18 Cystinosis
19 Dystrophinopathy (including Duchenne/Becker muscular dystrophy)
20 Fabry disease
21 Familial dysautonomia
22 Familial Mediterranean fever

23 Fanconi anemia complementation group A
24 Fragile X syndrome
25 Galactosemia
26 Gaucher disease
27 GJB2-related DFNB1 nonsyndromic hearing loss and deafness
28 Glutaric acidemia type 1
29 Glycogen storage disease type Ia
30 HADHA-related disorders (including Long Chain 3-Hydroxyacyl-CoA Dehydrogenase Deficiency)
31 Hb beta chain-related hemoglobinopathy (including beta-thalassemia and Sickle Cell disease)
32 Hereditary fructose intolerance
33 Herlitz junctional epidermolysis bullosa, LAMB3-related
34 Hexosaminidase A deficiency (including Tay-Sachs disease)
35 Homocystinuria caused by cystathionine beta-synthase deficiency
36 Hypophosphatasia, autosomal recessive
37 Krabbe disease
38 LAMA2-related muscular dystrophy
39 Lipoamide dehydrogenase deficiency
40 Maple syrup urine disease type 1B
41 Medium chain acyl-CoA dehydrogenase deficiency
42 Metachromatic leukodystrophy
43 Methylmalonic aciduria and homocystinuria, cblC type
44 MKS1-related disorders
45 Mucopolysaccaridosis type II
46 Mucopolysaccharidosis type I (Hurler syndrome)
47 MUT-related methylmalonic acidemia
48 NEB-related nemaline myopathy
49 Niemann-Pick disease type C
50 Nijmegen breakage syndrome
51 Ornithine transcarbamylase deficiency
52 Pendred syndrome
53 PEX1-related Zellweger syndrome spectrum
54 Phenylalanine hydroxylase deficiency (including phenylketonuria)
55 PKHD1-related autosomal recessive polycystic kidney disease (also known as autosomal recessive polycystic kidney disease)
56 Polyglandular autoimmune syndrome type 1
57 Pompe disease
58 Primary carnitine deficiency
59 Primary hyperoxaluria type 1
60 Rhizomelic chondrodysplasia punctata type 1
61 Sandhoff disease
62 Short chain acyl-CoA dehydrogenase deficiency
63 Smith-Lemli-Opitz syndrome
64 Spinal muscular atrophy
65 Steroid-resistant nephrotic syndrome
66 Sulfate transporter-related osteochondrodysplasia
67 Tyrosinemia type I
68 USH2A-related disorders (including Usher syndrome type 2)
69 Usher syndrome type 3
70 Very long chain acyl-CoA dehydrogenase deficiency
71 Wilson disease
72 X-linked adrenoleukodystrophy
73 X-linked Alport syndrome
74 X-linked congenital adrenal hypoplasia
75 X-linked juvenile retinoschisis
76 X-linked myotubular myopathy
77 X-linked severe combined immunodeficiency
78 Waiting on results of diagnostic test
79 Other Condition (Please Type In The Other Condition)
80 The pregnancy was found not to be affected
81 Don't recall

Q2. Were you pregnant when you received your carrier screening results?
(Choose One Answer)

1 Yes
2 No
3 Prefer not to answer

"PREFER NOT TO ANSWER" BLOCK

If "Prefer not to answer" on Q2:
Q3. After receiving your carrier screening results, what option(s) did you pursue or are you planning to pursue in the future? (Choose All That Apply)

1 In vitro fertilization (IVF) with preimplantation genetic diagnosis (PGD)
2 Sperm or egg donation
3 Adoption
4 Prenatal diagnostic testing such as chorionic villus sampling (CVS) or amniocentesis
5 If or when pregnant, inform other doctors that the baby might be at risk for {condition(s) indicated in Q1}
6 No longer planning to get pregnant
7 Testing other children or family members for {condition(s) indicated in Q1}
9 Other (Please Type In The Other Option)
8 Not planning to pursue any alternative options

"NOT PREGNANT" BLOCK

If "No" on Q2:
Q4. When you received your carrier screening results, were you undergoing or planning to undergo in vitro fertilization (IVF)?
(Choose One Answer)

1 Yes
2 No

If "No" on Q2:
Q5. After receiving your carrier screening results, what option(s) did you pursue or are you planning to pursue in the future? (Choose All That Apply)

1 In vitro fertilization (IVF) with preimplantation genetic diagnosis (PGD)
2 Use of donor sperm or egg
3 Adoption
4 Prenatal diagnostic testing such as chorionic villus sampling (CVS) or amniocentesis
5 Once pregnant, inform other doctors that baby might be at risk for {condition(s) indicated in Q1}
6 No longer planning to get pregnant
7 Test children or other family members for {condition(s) indicated in Q1}
9 Other (Please Type In The Other Option)
8 Not planning to pursue any other options

"PREGNANT" BLOCK

If "Yes" on Q2:
For the next few questions, please answer based on the pregnancy during which you received your carrier screening results. We'll ask about other pregnancies later.

Q6. How many weeks was your pregnancy at the time you received your carrier screening results? (Choose One Answer)

1 0-13 weeks (first trimester)
2 14-26 weeks (second trimester)

3 27 weeks or more (third trimester)
4 Don't recall

Q7. After receiving your carrier screening results, did you pursue prenatal diagnostic testing such as chorionic villus sampling (CVS) or amniocentesis for {condition(s) indicated in Q1}? (Choose One Answer)

1 Yes
2 No

If "No" on Q7:
Q8: What are some of the reasons you chose not to pursue prenatal diagnostic testing? (Please Type Your Answers In The Box Below. Please Be Specific And Include Any Details You Feel Comfortable Sharing.)

If "No" on Q7:
Q9: What was the outcome of the pregnancy? (Choose One Answer)

1 The pregnancy was continued and resulted in a live birth
2 The pregnancy was continued and the baby hasn't been born yet
3 The pregnancy was continued and was stillborn
4 The pregnancy miscarried
5 The pregnancy was terminated

Q10: If "The pregnancy was continued and resulted in a live birth" on Q9: Was the baby tested after he or she was born for {condition(s) indicated in Q1}? (Choose One Answer)

1 Yes, the baby was tested
2 No, but we plan to have the baby tested in the future
3 No, and we do not currently plan to have the baby tested

If "Yes" on Q7:
Q11: Which of the following condition(s) did the prenatal diagnostic test show that your pregnancy was affected with? (Choose All That Apply)

1 Alpha thalassemia
2 Aspartylglycosaminuria
3 Ataxia-telangiectasia
4 ATP7A-related disorders (including Menkes syndrome and Occipital Horn syndrome)
5 Bardet-Biedl syndrome, BBS1-related
6 Bardet-Biedl syndrome, BBS10-related
7 Biotinidase deficiency
8 Canavan disease
9 Carnitine palmitoyltransferase IA deficiency
10 Carnitine palmitoyltransferase II deficiency
11 Citrullinemia type 1
12 COL4A4-related Alport syndrome
13 Congenital adrenal hyperplasia (classic or non-classic)
14 Congenital disorder of glycosylation type Ia
15 Congenital disorder of glycosylation type Ic
16 Congenital Finnish nephrosis
17 Cystic fibrosis
18 Cystinosis
19 Dystrophinopathy (including Duchenne/Becker muscular dystrophy)
20 Fabry disease
21 Familial dysautonomia
22 Familial Mediterranean fever

23 Fanconi anemia complementation group A
24 Fragile X syndrome
25 Galactosemia
26 Gaucher disease
27 GJB2-related DFNB1 nonsyndromic hearing loss and deafness
28 Glutaric acidemia type 1
29 Glycogen storage disease type Ia
30 HADHA-related disorders (including Long Chain 3-Hydroxyacyl-CoA Dehydrogenase Deficiency)
31 Hb beta chain-related hemoglobinopathy (including beta-thalassemia and Sickle Cell disease)
32 Hereditary fructose intolerance
33 Herlitz junctional epidermolysis bullosa, LAMB3-related
34 Hexosaminidase A deficiency (including Tay-Sachs disease)
35 Homocystinuria caused by cystathionine beta-synthase deficiency
36 Hypophosphatasia, autosomal recessive
37 Krabbe disease
38 LAMA2-related muscular dystrophy
39 Lipoamide dehydrogenase deficiency
40 Maple syrup urine disease type 1B
41 Medium chain acyl-CoA dehydrogenase deficiency
42 Metachromatic leukodystrophy
43 Methylmalonic aciduria and homocystinuria, cblC type
44 MKS1-related disorders
45 Mucopolysaccaridosis type II
46 Mucopolysaccharidosis type I (Hurler syndrome)
47 MUT-related methylmalonic acidemia
48 NEB-related nemaline myopathy
49 Niemann-Pick disease type C
50 Nijmegen breakage syndrome
51 Ornithine transcarbamylase deficiency
52 Pendred syndrome
53 PEX1-related Zellweger syndrome spectrum
54 Phenylalanine hydroxylase deficiency (including phenylketonuria)
55 PKHD1-related autosomal recessive polycystic kidney disease (also known as autosomal recessive polycystic kidney disease)
56 Polyglandular autoimmune syndrome type 1
57 Pompe disease
58 Primary carnitine deficiency
59 Primary hyperoxaluria type 1
60 Rhizomelic chondrodysplasia punctata type 1
61 Sandhoff disease
62 Short chain acyl-CoA dehydrogenase deficiency
63 Smith-Lemli-Opitz syndrome
64 Spinal muscular atrophy
65 Steroid-resistant nephrotic syndrome
66 Sulfate transporter-related osteochondrodysplasia
67 Tyrosinemia type I
68 USH2A-related disorders (including Usher syndrome type 2)
69 Usher syndrome type 3
70 Very long chain acyl-CoA dehydrogenase deficiency
71 Wilson disease
72 X-linked adrenoleukodystrophy
73 X-linked Alport syndrome
74 X-linked congenital adrenal hypoplasia
75 X-linked juvenile retinoschisis
76 X-linked myotubular myopathy
77 X-linked severe combined immunodeficiency
78 Waiting on results of diagnostic test
79 Other Condition (Please Type In The Other Condition)
80 The pregnancy was found not to be affected
81 Don't recall

If “Yes” on Q7:
Q12: What was the outcome of the pregnancy after learning the final test result? (Choose One Answer)

1 The pregnancy was continued and resulted in a live birth
2 The pregnancy was continued and the baby hasn’t been born yet
3 The pregnancy was continued and was stillborn
4 The pregnancy miscarried
5 The pregnancy was terminated

SUBSEQUENT PREGNANCIES BLOCK

Q13: How many times have you been pregnant since receiving your carrier screening results? (Choose One Answer)

0 0
1 1
2 2 or more

If “1” or “2 or more” on Q13:
For the next few questions, please answer based on the next pregnancy <u>after</u> you received your carrier screening results.

Q14: In the next pregnancy after you received your carrier screening results, did you undergo in vitro fertilization (IVF) with preimplantation genetic diagnosis (PGD)? (Choose One Answer)

1 Yes
2 No

If “1” or “2 or more” on Q13:
Q15: In the next pregnancy after you received your carrier screening results, did you pursue prenatal diagnostic testing such as chorionic villus sampling (CVS) or amniocentesis?
(Choose One Answer)

1 Yes
2 No

If “No” on Q15:
Q16: What were some of the reasons you chose not to pursue prenatal diagnostic testing? (Please Type Your Answers In The Box Below. Please Be Specific And Include Any Details You Feel Comfortable Sharing.)

If “No” on Q15:
Q17: What was the outcome of the pregnancy? (Choose One Answer)

1 The pregnancy was continued and resulted in a live birth
2 The pregnancy was continued and the baby hasn’t been born yet
3 The pregnancy was continued and was stillborn
4 The pregnancy miscarried
5 The pregnancy was terminated

If “The pregnancy was continued and resulted in a live birth” on Q17:
Q18: Was the baby tested after he or she was born for {condition(s) indicated in Q1}?
(Choose One Answer)

1 Yes, the baby was tested
2 No, but we plan to have the baby tested in the future

3 No, and we do not currently plan to have the baby tested

If “Yes” on Q15:
Q19: Which of the following condition(s) did the final test show that your pregnancy was affected with? (Choose All That Apply)

1 Alpha thalassemia
2 Aspartylglycosaminuria
3 Ataxia-telangiectasia
4 ATP7A-related disorders (including Menkes syndrome and Occipital Horn syndrome)
5 Bardet-Biedl syndrome, BBS1-related
6 Bardet-Biedl syndrome, BBS10-related
7 Biotinidase deficiency
8 Canavan disease
9 Carnitine palmitoyltransferase IA deficiency
10 Carnitine palmitoyltransferase II deficiency
11 Citrullinemia type 1
12 COL4A4-related Alport syndrome
13 Congenital adrenal hyperplasia (classic or non-classic)
14 Congenital disorder of glycosylation type Ia
15 Congenital disorder of glycosylation type Ic
16 Congenital Finnish nephrosis
17 Cystic fibrosis
18 Cystinosis
19 Dystrophinopathy (including Duchenne/Becker muscular dystrophy)
20 Fabry disease
21 Familial dysautonomia
22 Familial Mediterranean fever
23 Fanconi anemia complementation group A
24 Fragile X syndrome
25 Galactosemia
26 Gaucher disease
27 GJB2-related DFNB1 nonsyndromic hearing loss and deafness
28 Glutaric acidemia type 1
29 Glycogen storage disease type Ia
30 HADHA-related disorders (including Long Chain 3-Hydroxyacyl-CoA Dehydrogenase Deficiency)
31 Hb beta chain-related hemoglobinopathy (including beta-thalassemia and Sickle Cell disease)
32 Hereditary fructose intolerance
33 Herlitz junctional epidermolysis bullosa, LAMB3-related
34 Hexosaminidase A deficiency (including Tay-Sachs disease)
35 Homocystinuria caused by cystathionine beta-synthase deficiency
36 Hypophosphatasia, autosomal recessive
37 Krabbe disease
38 LAMA2-related muscular dystrophy
39 Lipoamide dehydrogenase deficiency
40 Maple syrup urine disease type 1B
41 Medium chain acyl-CoA dehydrogenase deficiency
42 Metachromatic leukodystrophy
43 Methylmalonic aciduria and homocystinuria, cblC type
44 MKS1-related disorders
45 Mucopolysaccaridosis type II
46 Mucopolysaccharidosis type I (Hurler syndrome)
47 MUT-related methylmalonic acidemia
48 NEB-related nemaline myopathy
49 Niemann-Pick disease type C
50 Nijmegen breakage syndrome
51 Ornithine transcarbamylase deficiency
52 Pendred syndrome
53 PEX1-related Zellweger syndrome spectrum
54 Phenylalanine hydroxylase deficiency (including phenylketonuria)

55 PKHD1-related autosomal recessive polycystic kidney disease (also known as autosomal recessive polycystic kidney disease)
56 Polyglandular autoimmune syndrome type 1
57 Pompe disease
58 Primary carnitine deficiency
59 Primary hyperoxaluria type 1
60 Rhizomelic chondrodysplasia punctata type 1
61 Sandhoff disease
62 Short chain acyl-CoA dehydrogenase deficiency
63 Smith-Lemli-Opitz syndrome
64 Spinal muscular atrophy
65 Steroid-resistant nephrotic syndrome
66 Sulfate transporter-related osteochondrodysplasia
67 Tyrosinemia type I
68 USH2A-related disorders (including Usher syndrome type 2)
69 Usher syndrome type 3
70 Very long chain acyl-CoA dehydrogenase deficiency
71 Wilson disease
72 X-linked adrenoleukodystrophy
73 X-linked Alport syndrome
74 X-linked congenital adrenal hypoplasia
75 X-linked juvenile retinoschisis
76 X-linked myotubular myopathy
77 X-linked severe combined immunodeficiency
78 Waiting on results of diagnostic test
79 Other Condition (Please Type In The Other Condition)
80 The pregnancy was found not to be affected
81 Don't recall

If "Yes" on Q15:
Q20: What was the outcome of the pregnancy after learning the final test result? (Choose One Answer)

1 The pregnancy was continued and resulted in a live birth
2 The pregnancy was continued and the baby hasn't been born yet
3 The pregnancy was continued and was stillborn
4 The pregnancy miscarried
5 The pregnancy was terminated

If "2 or more" on Q13:
Q21: In your most recent pregnancy, did you undergo in vitro fertilization (IVF) with preimplantation genetic diagnosis (PGD)? (Choose One Answer)

1 Yes
2 No

If "2 or more" on Q13:
Q22: In your most recent pregnancy, did you pursue prenatal diagnostic testing such as chorionic villus sampling (CVS) or amniocentesis? (Choose One Answer)

1 Yes
2 No

If "No" on Q22:
Q23: What were some of the reasons you chose not to pursue prenatal diagnostic testing? (Please Type Your Answers In The Box Below. Please Be Specific And Include Any Details You Feel Comfortable Sharing.)

If “No” on Q22:
Q24: What was the outcome of the pregnancy? (Choose One Answer)

1 The pregnancy was continued and resulted in a live birth
2 The pregnancy was continued and the baby hasn’t been born yet
3 The pregnancy was continued and was stillborn
4 The pregnancy miscarried
5 The pregnancy was terminated

If “The pregnancy was continued and resulted in a live birth” on Q24:
Q25: Was the baby tested after he or she was born for {condition(s) indicated in Q1}? (Choose One Answer)

1 Yes, the baby was tested
2 No, but we plan to have the baby tested in the future
3 No, and we do not currently plan to have the baby tested

If “Yes” on Q22:
Q26: Which of the following condition(s) did the final test show that your pregnancy was affected with? (Choose All That Apply)

1 Alpha thalassemia
2 Aspartylglycosaminuria
3 Ataxia-telangiectasia
4 ATP7A-related disorders (including Menkes syndrome and Occipital Horn syndrome)
5 Bardet-Biedl syndrome, BBS1-related
6 Bardet-Biedl syndrome, BBS10-related
7 Biotinidase deficiency
8 Canavan disease
9 Carnitine palmitoyltransferase IA deficiency
10 Carnitine palmitoyltransferase II deficiency
11 Citrullinemia type 1
12 COL4A4-related Alport syndrome
13 Congenital adrenal hyperplasia (classic or non-classic)
14 Congenital disorder of glycosylation type Ia
15 Congenital disorder of glycosylation type Ic
16 Congenital Finnish nephrosis
17 Cystic fibrosis
18 Cystinosis
19 Dystrophinopathy (including Duchenne/Becker muscular dystrophy)
20 Fabry disease
21 Familial dysautonomia
22 Familial Mediterranean fever
23 Fanconi anemia complementation group A
24 Fragile X syndrome
25 Galactosemia
26 Gaucher disease
27 GJB2-related DFNB1 nonsyndromic hearing loss and deafness
28 Glutaric acidemia type 1
29 Glycogen storage disease type Ia
30 HADHA-related disorders (including Long Chain 3-Hydroxyacyl-CoA Dehydrogenase Deficiency)
31 Hb beta chain-related hemoglobinopathy (including beta-thalassemia and Sickle Cell disease)
32 Hereditary fructose intolerance
33 Herlitz junctional epidermolysis bullosa, LAMB3-related
34 Hexosaminidase A deficiency (including Tay-Sachs disease)
35 Homocystinuria caused by cystathionine beta-synthase deficiency
36 Hypophosphatasia, autosomal recessive
37 Krabbe disease
38 LAMA2-related muscular dystrophy
39 Lipoamide dehydrogenase deficiency

40 Maple syrup urine disease type 1B
41 Medium chain acyl-CoA dehydrogenase deficiency
42 Metachromatic leukodystrophy
43 Methylmalonic aciduria and homocystinuria, cblC type
44 MKS1-related disorders
45 Mucopolysaccaridosis type II
46 Mucopolysaccharidosis type I (Hurler syndrome)
47 MUT-related methylmalonic acidemia
48 NEB-related nemaline myopathy
49 Niemann-Pick disease type C
50 Nijmegen breakage syndrome
51 Ornithine transcarbamylase deficiency
52 Pendred syndrome
53 PEX1-related Zellweger syndrome spectrum
54 Phenylalanine hydroxylase deficiency (including phenylketonuria)
55 PKHD1-related autosomal recessive polycystic kidney disease (also known as autosomal recessive polycystic kidney disease)
56 Polyglandular autoimmune syndrome type 1
57 Pompe disease
58 Primary carnitine deficiency
59 Primary hyperoxaluria type 1
60 Rhizomelic chondrodysplasia punctata type 1
61 Sandhoff disease
62 Short chain acyl-CoA dehydrogenase deficiency
63 Smith-Lemli-Opitz syndrome
64 Spinal muscular atrophy
65 Steroid-resistant nephrotic syndrome
66 Sulfate transporter-related osteochondrodysplasia
67 Tyrosinemia type I
68 USH2A-related disorders (including Usher syndrome type 2)
69 Usher syndrome type 3
70 Very long chain acyl-CoA dehydrogenase deficiency
71 Wilson disease
72 X-linked adrenoleukodystrophy
73 X-linked Alport syndrome
74 X-linked congenital adrenal hypoplasia
75 X-linked juvenile retinoschisis
76 X-linked myotubular myopathy
77 X-linked severe combined immunodeficiency
78 Waiting on results of diagnostic test
79 Other Condition (Please Type In The Other Condition)
80 The pregnancy was found not to be affected
81 Don't recall

If “Yes” on Q22:
Q27. What was the outcome of the pregnancy after learning the final test result?
(Choose One Answer)

1 The pregnancy was continued and resulted in a live birth
2 The pregnancy was continued and the baby hasn’t been born yet
3 The pregnancy was continued and was stillborn
4 The pregnancy miscarried
5 The pregnancy was terminated

DEMOGRAPHIC SECTION

Q28: What was the age of the female partner at the time of her original carrier screening? (Choose One Answer)

1 Younger than 18

2	18-24
3	25-34
4	35-44
5	45 or over

Q29: What is the female partner’s ethnicity? (Choose All That Apply)

1	Northern European (e.g., British, German)
2	Southern European (e.g., Italian, Greek)
3	French Canadian or Cajun
4	Ashkenazi Jewish
5	Other/Mixed Caucasian
6	East Asian (e.g., Chinese, Japanese)
7	South Asian (e.g., Indian, Pakistani)
8	Southeast Asian (e.g., Filipino, Vietnamese)
9	African or African American
10	Hispanic
11	Middle Eastern
12	Native American
13	Pacific Islander
14	Other (Please Type In The Other Ethnicity)
15	Unknown
16	Prefer not to say

Q30: What is the male partner’s ethnicity?
(Choose All That Apply)

1	Northern European (e.g., British, German)
2	Southern European (e.g., Italian, Greek)
3	French Canadian or Cajun
4	Ashkenazi Jewish
5	Other/Mixed Caucasian
6	East Asian (e.g., Chinese, Japanese)
7	South Asian (e.g., Indian, Pakistani)
8	Southeast Asian (e.g., Filipino, Vietnamese)
9	African or African American
10	Hispanic
11	Middle Eastern
12	Native American
13	Pacific Islander
14	Other (Please Type In The Other Ethnicity)
15	Unknown
16	Prefer not to say

Q31: In what state did you live when you received your carrier screening results?
(Choose One Answer from drop-down list. Includes “Outside the U.S. option.)

Q32. What is the female partner’s religious affiliation? (Choose One Answer)

1	Protestant
2	Catholic
3	Mormon
4	Jewish
5	Buddhist
6	Hindu
7	Muslim
8	Agnostic
9	Atheist

10 Other (Please Type In The Other Religion)
11 No religious affiliation
12 Unknown
13 Prefer not to say

Q33. What is the male partner's religious affiliation? (Choose One Answer)

1 Protestant
2 Catholic
3 Mormon
4 Jewish
5 Buddhist
6 Hindu
7 Muslim
8 Agnostic
9 Atheist
10 Other (Please Type In The Other Religion)
11 No religious affiliation
12 Unknown
13 Prefer not to say
