# Supplementary material for: Clinical utility of expanded carrier screening: results-guided actionability and outcomes
Source: Genet Med. 2018 Oct 11;21(5):1041–8. doi: 10.1038/s41436-018-0321-0 (PMC6752268; doi:10.1038/s41436-018-0321-0)
Supplement: Supplementary file 2 — Supplementary Table 1 [file 41436_2018_321_MOESM2_ESM.pdf]

Supplementary Table 1. Conditions reported by respondents, categorized by severity[a].

| Condition(s) reported[b] | n (%) |
|---|---|
| Profound | 50 (13) |
| Smith-Lemli-Opitz syndrome | 6 (1.5) |
| Smith-Lemli-Opitz syndrome, Fragile X syndrome | 4 (1.0) |
| Hexosaminidase A deficiency (Tay-Sachs disease) | 4 (1.0) |
| Congenital disorder of glycosylation type Ia | 3 (0.8) |
| Pompe disease | 3 (0.8) |
| Gaucher disease | 2 (0.5) |
| Gaucher disease, Fragile X syndrome | 2 (0.5) |
| Niemann-Pick disease type C | 2 (0.5) |
| Canavan disease | 1 (0.2) |
| Canavan disease, Fragile X syndrome | 1 (0.2) |
| Congenital disorder of glycosylation type Ia, Biotinidase deficiency, Cystic fibrosis | 1 (0.2) |
| Congenital disorder of glycosylation type Ic | 1 (0.2) |
| Familial dysautonomia, Fragile X syndrome | 1 (0.2) |
| Galactosemia | 1 (0.2) |
| Galactosemia, Fragile X syndrome | 1 (0.2) |
| Gaucher disease, Familial Mediterranean fever | 1 (0.2) |
| Gaucher disease, Biotinidase deficiency, Pendred syndrome | 1 (0.2) |
| HADHA-related disorders (including Long Chain 3-Hydroxyacyl-CoA Dehydrogenase Deficiency) | 1 (0.2) |
| Hexosaminidase A deficiency (Tay-Sachs disease), Fragile X syndrome | 1 (0.2) |
| Krabbe disease | 1 (0.2) |
| Krabbe disease, GJB2-related DFNB1 nonsyndromic hearing loss and deafness | 1 (0.2) |
| Lipoamide dehydrogenase deficiency | 1 (0.2) |
| Maple syrup urine disease type 1B, Fragile X syndrome | 1 (0.2) |
| Maple syrup urine disease type 1B, Fragile X syndrome, Familial Mediterranean Fever | 1 (0.2) |
| Medium chain acyl-CoA dehydrogenase deficiency | 1 (0.2) |
| Medium chain acyl-CoA dehydrogenase deficiency, Fragile X syndrome | 1 (0.2) |
| Metachromatic leukodystrophy | 1 (0.2) |
| Mucopolysaccharidosis type I (Hurler syndrome) | 1 (0.2) |
| PEX1-related Zellweger syndrome spectrum, Biotinidase deficiency | 1 (0.2) |
| Rhizomelic chondrodysplasia punctata type 1 | 1 (0.2) |
| Tyrosinemia type I | 1 (0.2) |
| X-linked adrenoleukodystrophy | 1 (0.2) |
| Severe | 258 (66) |
| Fragile X syndrome | 128 (33) |
| Cystic fibrosis | 38 (10) |
| Familial Mediterranean fever | 9 (2.3) |
| Dystrophinopathy (including Duchenne/Becker muscular dystrophy) | 8 (2.0) |
| Spinal muscular atrophy | 8 (2.0) |
| Alpha thalassemia | 7 (1.8) |
| Phenylalanine hydroxylase deficiency | 5 (1.3) |
| Fabry disease | 4 (1.0) |
| Hb beta chain-related hemoglobinopathy (including Beta-thalassemia and Sickle Cell disease) | 4 (1.0) |
| Wilson disease | 3 (0.8) |
| PKHD1-related autosomal recessive polycystic kidney disease | 2 (0.5) |
| Alpha thalassemia, Congenital adrenal hyperplasia | 2 (0.5) |
| Cystic fibrosis, Fragile X syndrome | 2 (0.5) |
| Fragile X syndrome, Congenital adrenal hyperplasia | 2 (0.5) |
| Fragile X syndrome, Spinal muscular atrophy | 2 (0.5) |
| Alpha thalassemia, Biotinidase deficiency | 1 (0.2) |
| Alpha thalassemia, Congenital adrenal hyperplasia, Fragile X syndrome | 1 (0.2) |

| | |
|---|---|
| Alpha thalassemia, Fragile X syndrome | 1 (0.2) |
| Alpha thalassemia, Hb beta chain-related hemoglobinopathy (including Beta-thalassemia and Sickle Cell disease) | 1 (0.2) |
| Alpha thalassemia, Phenylalanine hydroxylase deficiency | 1 (0.2) |
| Ataxia-telangiectasia | 1 (0.2) |
| Bardet-Biedl syndrome BBS10-related, Cystic fibrosis | 1 (0.2) |
| Biotinidase deficiency | 1 (0.2) |
| Cystic fibrosis, GJB2-related DFNB1 nonsyndromic hearing loss and deafness | 1 (0.2) |
| Cystic fibrosis, Congenital adrenal hyperplasia | 1 (0.2) |
| Cystic fibrosis, Hereditary fructose intolerance | 1 (0.2) |
| Cystic fibrosis, Fragile X syndrome, Spinal muscular atrophy | 1 (0.2) |
| Cystic fibrosis, Spinal muscular atrophy | 1 (0.2) |
| Dystrophinopathy (including Duchenne/Becker muscular dystrophy), Glycogen storage disease type Ia | 1 (0.2) |
| Dystrophinopathy (including Duchenne/Becker muscular dystrophy), Spinal muscular atrophy | 1 (0.2) |
| Familial Mediterranean fever, Fragile X syndrome | 1 (0.2) |
| Familial Mediterranean fever, GJB2-related DFNB1 nonsyndromic hearing loss and deafness | 1 (0.2) |
| Fanconi anemia complementation group A | 1 (0.2) |
| Fragile X syndrome, GJB2-related DFNB1 nonsyndromic hearing loss and deafness | 1 (0.2) |
| Fragile X syndrome, Herlitz junctional epidermolysis bullosa LAMB3-related, GJB2-related DFNB1 nonsyndromic hearing loss and deafness | 1 (0.2) |
| Fragile X syndrome, Phenylalanine hydroxylase deficiency | 1 (0.2) |
| Fragile X syndrome, Wilson disease | 1 (0.2) |
| Glycogen storage disease type Ia | 1 (0.2) |
| Hereditary fructose intolerance, Congenital adrenal hyperplasia | 1 (0.2) |
| Herlitz junctional epidermolysis bullosa LAMB3-related, Primary hyperoxaluria type 1, Spinal muscular atrophy | 1 (0.2) |
| Homocystinuria caused by cystathionine beta-synthase deficiency | 1 (0.2) |
| Hypophosphatasia autosomal recessive | 1 (0.2) |
| LAMA2-related muscular dystrophy | 1 (0.2) |
| Nijmegen breakage syndrome | 1 (0.2) |
| Spinal muscular atrophy, GJB2-related DFNB1 nonsyndromic hearing loss and deafness | 1 (0.2) |
| Sulfate transporter-related osteochondrodysplasia | 1 (0.2) |
| Usher syndrome type | 1 (0.2) |
| X-linked Alport syndrome | 1 (0.2) |
| X-linked congenital adrenal hypoplasia | 1 (0.2) |
| Moderate | 62 (16) |
| GJB2-related DFNB1 nonsyndromic hearing loss and deafness | 32 (8.2) |
| Congenital adrenal hyperplasia | 25 (6.4) |
| Pendred syndrome | 4 (1.0) |
| X-linked juvenile retinoschisis | 1 (0.2) |
| Don't recall | 17 (3.6) |
| None | 2 (0.5) |
| Answer unclear | 2 (0.5) |
| **Total Respondents** | **391 (100)** |

a. Conditions were placed into severity categories according to the method described by Lazarin et al.[7] Briefly, conditions categorized as Profound are those that result in a shortened lifespan (infancy or childhood/adolescence) and/or intellectual disability; severe conditions are those that result in a shortened lifespan (premature adulthood), impaired mobility, and/or internal physical malformation; and moderate conditions are those that result in sensory impairment (vision, hearing, touch or other), immunodeficiency, cancer, mental illness, and/or dysmorphic features.

b. Percents sum to just over 100% due to rounding. Conditions listed on the same row and separated by commas represent participants reporting that their current or future pregnancies were at risk for both or all three conditions.
