# Supplementary material for: Clinical utility of expanded carrier screening: results-guided actionability and outcomes
Source: Genet Med. 2018 Oct 11;21(5):1041–8. doi: 10.1038/s41436-018-0321-0 (PMC6752268; doi:10.1038/s41436-018-0321-0)
Supplement: Supplementary file 3 — Supplementary Table 2 [file 41436_2018_321_MOESM3_ESM.pdf]

Supplementary Table 2. Outcomes of pregnancies found by PNDx to be affected, with condition listed.

	Total pregnancies, n	Pregnancy during which ECS results were received, n	Pregnancies conceived after ECS results were received, n
Affected pregnancies	32	20	12
Terminated (%)	17 (53)	8 (40)	9 (75)
Congenital adrenal hyperplasia	1	-	1
Cystic fibrosis	4	2	2
Fragile X syndrome	2	1	1
GJB2-related DFNB1 nonsyndromic hearing loss and deafness	2	1	1
HADHA-related disorders (including Long Chain 3-Hydroxyacyl-CoA Dehydrogenase Deficiency)	1	1	-
Hypophosphatasia (autosomal recessive)	1	-	1
LAMA2-related muscular dystrophy	1	1	-
Niemann-Pick disease type C	2	1	1
Phenylalanine hydroxylase deficiency	1	1	-
Pompe disease	1	-	1
Spinal muscular atrophy	2	-	2
Live birth (%)	11 (34)	8 (40)	3 (25)
Cystic fibrosis	1	1	-
Congenital adrenal hyperplasia	1	1	-
Congenital adrenal hypoplasia	1	1	-
Fragile X syndrome	5	2	3
GJB2-related DFNB1 nonsyndromic hearing loss and deafness	1	1	-
Pompe disease	1	1	-
Spinal muscular atrophy	1	1	-
Not born yet (%)	3 (9.4)	3 (15)	0 (0)
Congenital adrenal hyperplasia	1	1	-
Medium chain acyl-CoA dehydrogenase deficiency	1	1	-
Unknown[a]	1	1	
Stillborn (%)	1 (3.1)	1 (5.0)	0 (0)
PKHD1-related autosomal recessive polycystic kidney disease	1	1	-
Miscarried (%)	0 (0)	0 (0)	0 (0)

a. 1 at-risk couple (ARC) could not recall the condition for which the pregnancy was found to be affected.
