# Supplementary material for: Clinical utility of expanded carrier screening: results-guided actionability and outcomes
Source: Genet Med. 2018 Oct 11;21(5):1041–8. doi: 10.1038/s41436-018-0321-0 (PMC6752268; doi:10.1038/s41436-018-0321-0)
Supplement: Supplementary file 4 — Supplementary Table 3 [file 41436_2018_321_MOESM4_ESM.pdf]

Supplementary Table 3. Reasons cited by ARCs choosing not to undergo prenatal diagnosis.

	All pregnancies, n (%; CI)	Pregnancy during screening, n (%; CI)	Subsequent pregnancy, n (%; CI)
Did not undergo prenatal diagnosis	213 (100)	95 (100)	118 (100)
Reason[a]			
Risk of miscarriage	50 (23; 18-29)	33 (35; 26-45)[b]	17 (14; 9-22)[b]
Perception of low risk of pregnancy being affected	45 (21; 16-27)	25 (26; 18-36)	20 (17; 11-24)
Would not have terminated the pregnancy	38 (18; 13-23)	26 (27; 19-37)[c]	12 (10; 6-17)[c]
Not necessary because IVF with PGT-M was performed	31 (15; 11-21)	0 (0; 0-3)	31 (28; 20-37)
Pregnancy miscarried before testing could be performed	16 (8; 5-12)	0 (0; 0-3)	16 (14; 8-21)
Condition phenotype not severe enough to warrant testing	12 (6; 3-10)	6 (6.3; 3-13)	6 (5.1; 2-10)
Planning post-natal testing instead	8 (4; 2-7)	5 (5.3; 2-11)	3 (2.5; 1-7)
No prenatal treatment options available	4 (1.9; 1-4)	3 (3.2; 1-8)	1 (0.8; 0-4)
Too late in the pregnancy to terminate	4 (1.9; 1-4)	3 (3.2; 1-8)	1 (0.8; 0-4)
Provider did not recommend testing	4 (1.9; 1-4)	2 (2.1; 0-7)	2 (1.7; 0-5)
Cost was too high	3 (1.4; 0-4)	1 (1.1; 0-5)	2 (1.7; 0-5)
Pregnancy was terminated before pursuing testing	2 (0.9; 0-3)	0 (0; 0-3)	2 (1.7; 0-5)
Too early in pregnancy to undergo testing	2 (0.9; 0-3)	0 (0; 0-3)	2 (1.7; 0-5)
Other/Did not specify	24 (11; 8-26)	7 (7.4; 3-14)	17 (14; 9-22)

ARC: At-risk couple. CI: confidence interval, 95%. IVF: In vitro fertilization. PGT-M: Preimplantation genetic testing for monogenic conditions.

a. Percents will sum to greater than 100% because respondents could cite more than one reason.
b. Significantly different at the 95% confidence level (p=0.0005).
c. Significantly different at the 95% confidence level (p=0.001).
